# Supplementary material for: Queuosine is incorporated into precursor tRNA before splicing
Source: Nat Commun. 2025 Jul 31;16:7044. doi: 10.1038/s41467-025-62220-z (PMC12313893; doi:10.1038/s41467-025-62220-z)
Supplement: Supplementary file 1 — Supplementary Information [file 41467_2025_62220_MOESM1_ESM.pdf]

Supplementary Information

Queuosine is incorporated into precursor tRNA before splicing

Wei Guo[1,2,3*], Igor Kaczmarczyk[4,5*], Kevin Kopietz[6], Florian Flegler[7], Stefano Russo[1,2,3], Ege Cigirgan[8], Andrzej Chramiec-Głąbik[4], Łukasz Koziej[4], Cansu Cirzi[9], Jirka Peschek[8], Klaus Reuter[7], Mark Helm[6], Sebastian Glatt[4,10] and Francesca Tuorto[2,3§]

*Equal contribution

Affiliations

1. Faculty of Biosciences, Heidelberg University, 69120 Heidelberg, Germany.
2. Center for Molecular Biology of Heidelberg University (ZMBH), DKFZ-ZMBH Alliance, 69120 Heidelberg, Germany.
3. Division of Biochemistry, Mannheim Institute for Innate Immunoscience (MI3), Mannheim Cancer Center (MCC), Medical Faculty Mannheim, Heidelberg University, 68167 Mannheim, Germany.
4. Małopolska Centre of Biotechnology, Jagiellonian University, 30-387 Kraków, Poland.
5. Doctoral School of Exact and Natural Sciences, Jagiellonian University, Kraków, Poland
6. Institute of Pharmaceutical and Biomedical Science (IPBS), Johannes Gutenberg-University Mainz, 55128 Mainz, Germany.
7. Institut für Pharmazeutische Chemie, Philipps-Universität Marburg, 35037 Marburg, Germany.
8. Biochemistry Center (BZH), Heidelberg University, 69120 Heidelberg, Germany.
9. Division of Epigenetics, DKFZ-ZMBH Alliance, German Cancer Research Center (DKFZ), 69120 Heidelberg, Germany.
10. Department for Biological Sciences and Pathobiology, University of Veterinary Medicine Vienna, 1210 Vienna, Austria

[§] Corresponding author:
Phone: +49-621 38371439
Email: francesca.tuorto@medma.uni-heidelberg.de

Supplementary Figures

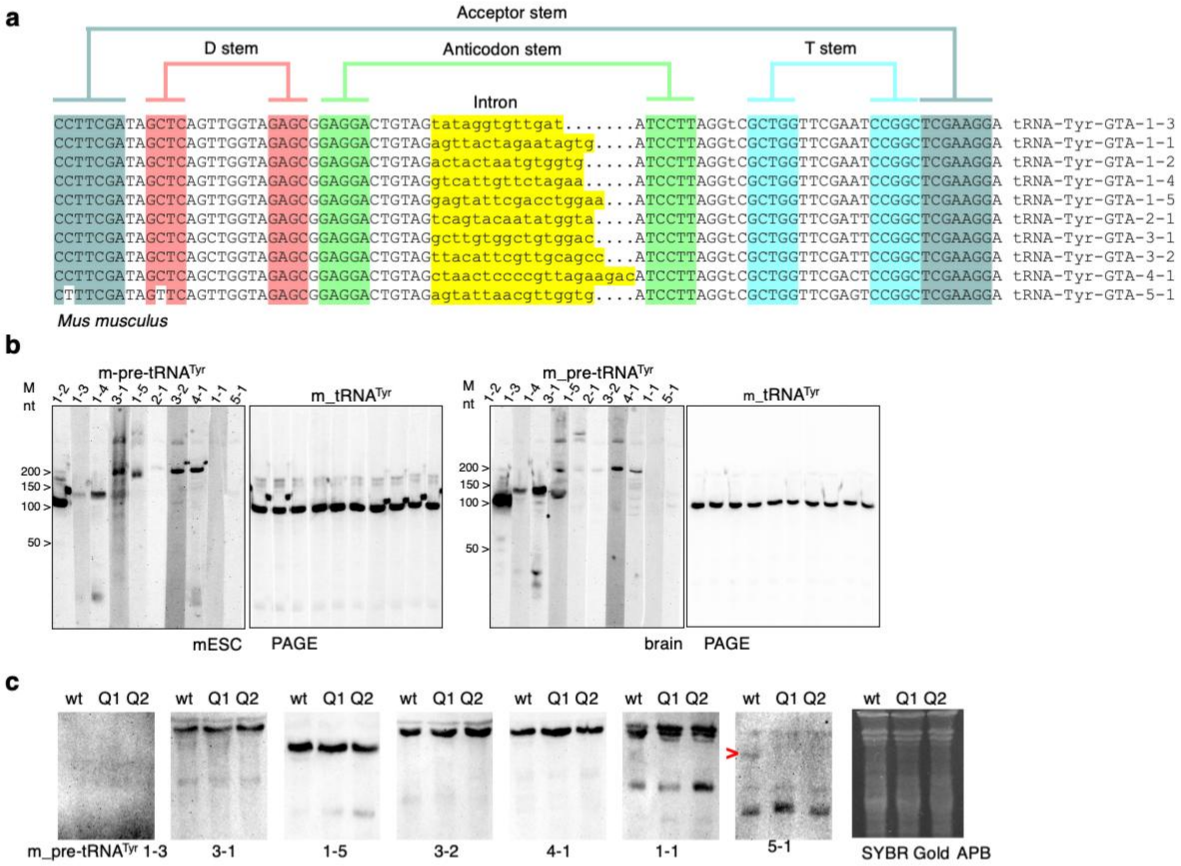

Supplementary Fig. 1. tRNA^{Tyr} is queuosinylated at precursor level in mESCs and tissues. **a** Sequences of all the mouse intron containing tRNA^{Tyr} according to gtRNA database (https://gtrnadb.ucsc.edu/genomes/eukaryota/Mmusc39/). Specific probes are designed to be reverse complementary to the intron sequence in yellow. **b** Semiquantitative Northern blot showing the expression of all ten pre-tRNA^{Tyr} in mESCs and brain. Each lane loaded with 10µg of total RNA is hybridized with the indicated pre-tRNA probe, and after detection, all the membranes are hybridized with tRNA^{Tyr} probe, which is used as loading control for the quantification (Fig.1d). **c** Systematic analysis of queuosinylation in pre-tRNA^{Tyr} using APB Northern blot in wild type mESCs. Q1 and Q2 knockout clones are shown as negative control. The red arrow indicates the shift produced by Q. Pre-tRNA^{Tyr} 1-4, 2-1 and1-2 are showed in the Fig.1f. wt: wild type, Q1: *Qtrt1*$^{-/-}$, Q2: *Qtrt2*$^{-/-}$, M: molecular weight marker, nt: nucleotides.

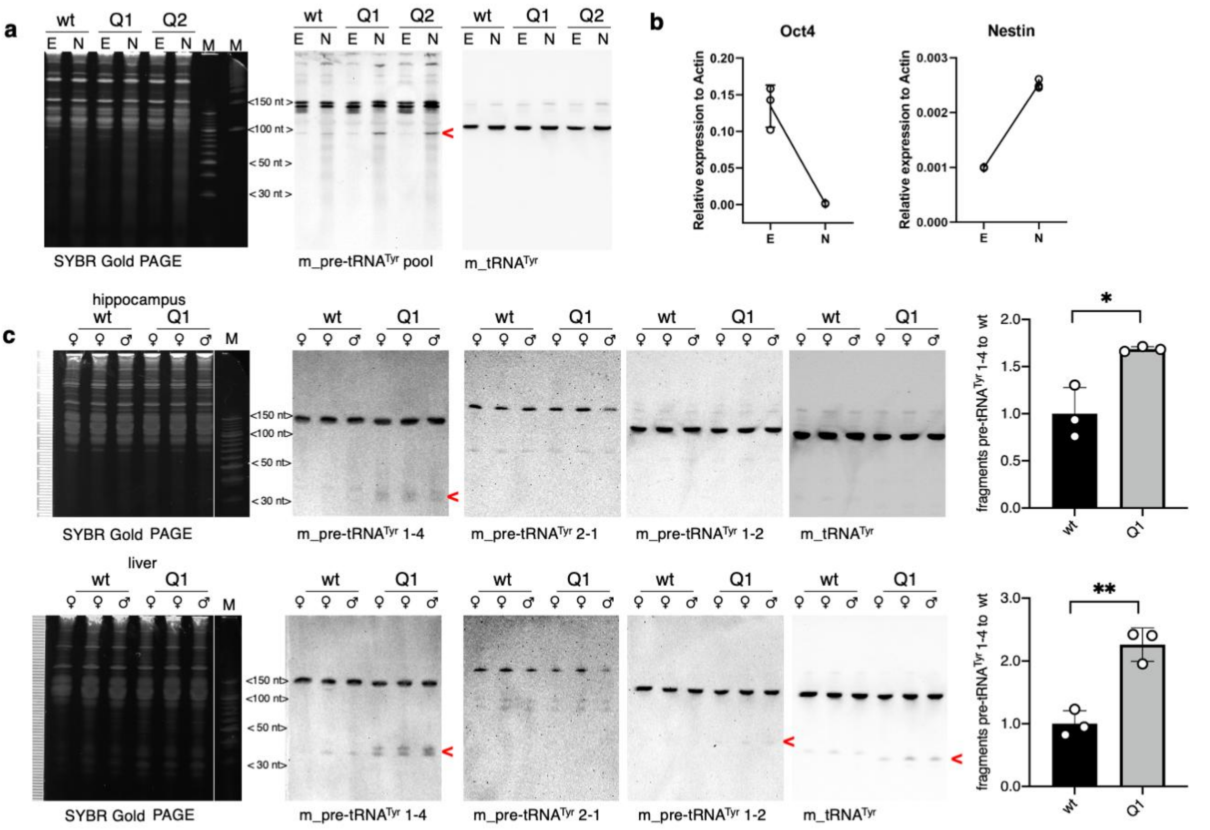

Supplementary Fig. 2. Q dependent pre-tRNA^{Tyr} stability in mESCs and tissues.
a No major differences in full length pre-tRNA^{Tyr} and mature tRNA^{Tyr} stability are detected using PAGE Northern blot in embryonic stem cells (E) and neuronal differentiated cells (N) upon Q loss. Increased pre-tRNA fragments are indicated by the red arrow. **b** Neuronal differentiation of mESCs is validated using qPCR of Oct4 and Nestin as markers of stemness and neuronal differentiation, respectively. Error bars represent ±SD (n=3, technical replicates). **c** Discrete pre-tRNA^{Tyr} probes in mouse hippocampus brain (upper panels) and liver (lower panels) using PAGE Northern blot show no genotypes related differences in expression. Increased pre-tRNA fragments in Q1 are indicated by the red arrows. Quantification of the fragments of pre-tRNA^{Tyr} 1-4 in Q1 normalized to the full-length pre-tRNA^{Tyr}1-4 and relative to wild type is provided for hippocampus (n=3, biological replicates) and liver (n=3, biological replicates). Error bars represent ±SD. *=p-value < 0.05, **=p-value < 0.01. Statistical significance was analyzed by two-tailed unpaired t-test. wt: wild type, Q: *Qtrt1*$^{-/-}$, M: molecular weight marker, nt: nucleotides.

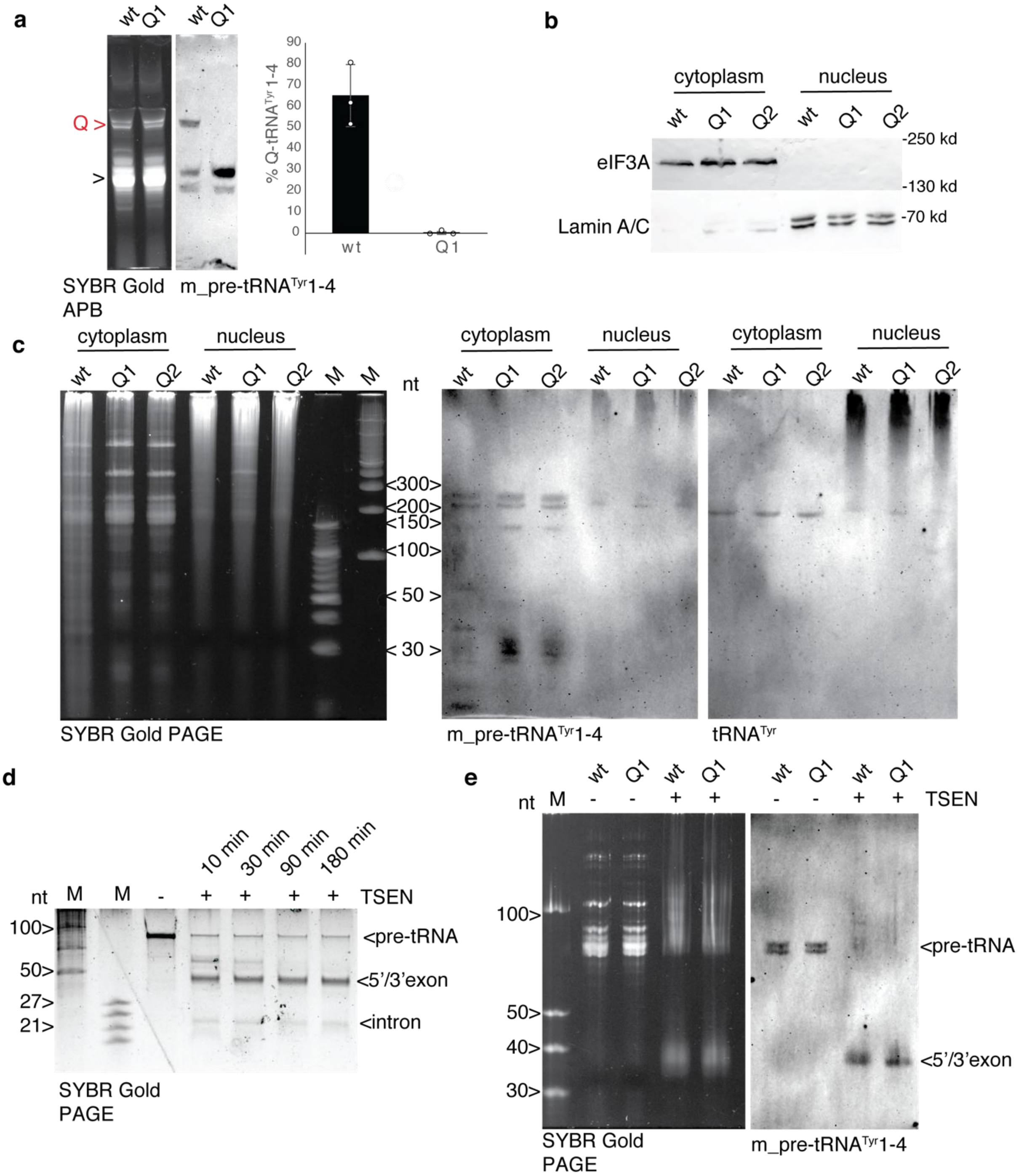

Supplementary Fig. 3. Cellular fractionation and splicing assay.
a APB Northern blotting and quantification of queuosinylation of pre-tRNA^{Tyr} 1-4 (65% of total) in mESCs used in **b**, **c** and **d**. (n=3, biological replicates, error bars represent standard deviation). The red arrow indicates the shift produced by Q, the black arrow indicates unmodified pre-tRNA. **b** Western blot of the indicated cellular fractions. eIF3A and LAMIN A/C are used as markers of cytoplasm and nuclear fraction respectively. **c** APB Northern blotting of the samples indicated in **b** showing the expression of pre-tRNA^{Tyr} 1-4 in the respective in cytoplasm/nuclear fractions. **d** Cleavage of *in vitro* transcribed human pre-tRNA$^{Tyr}{}_{GUA}$ 1-1 using recombinant TSEN complex. **e** PAGE Northern blotting with a probe targeting pre-tRNA^{Tyr} 1-4 shows the same cutting efficiency by TSEN on bulk tRNA isolated from Q1 and wild type mESCs. Q: queuosine, wt: wild type, Q: *Qtrt1*$^{-/-}$, M: molecular weight marker, nt: nucleotides.

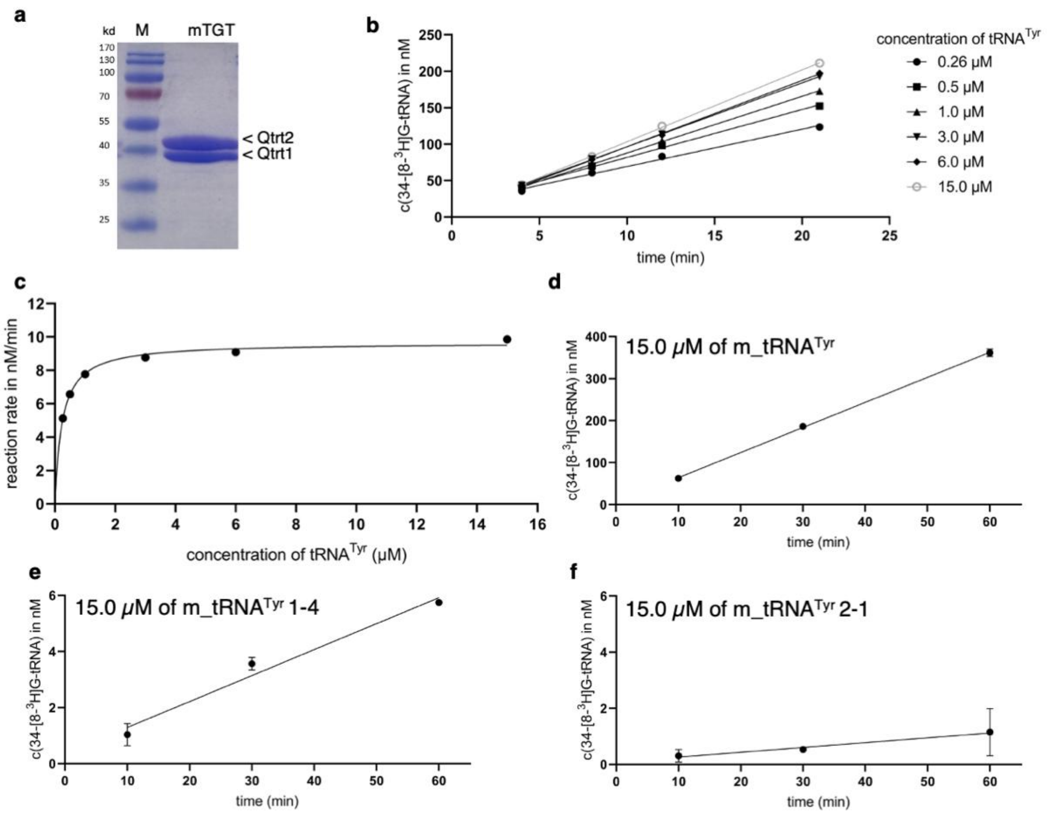

g

	Reaction rate (v_0)	K_{cat}
m_tRNA^{Tyr}	6,11 ± 0,39 nM/min	0,061 ± 0,004 1/min
m_pre-tRNA^{Tyr} 1-4	0,085 ± 0,022 nM/min	0,00085 ± 0,00022 1/min
m_pre-tRNA^{Tyr} 2-1	0,017 ± 0,019 nM/min	0,000035 ± 0,000190 1/min

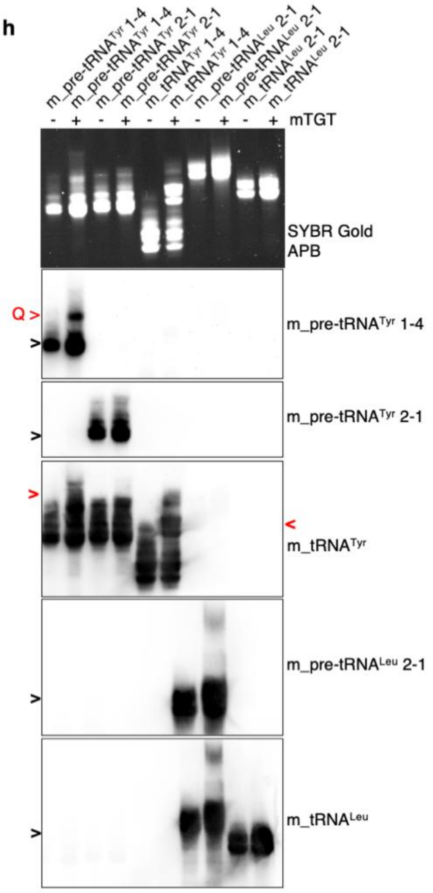

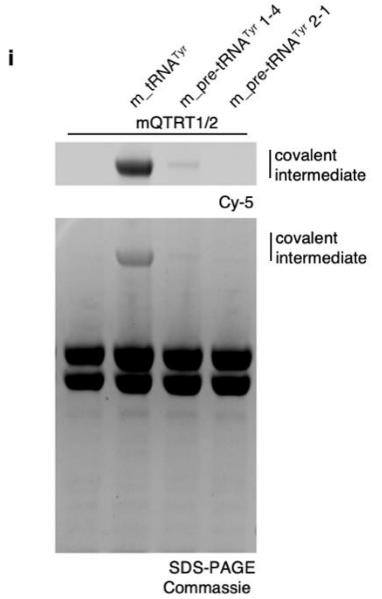

Supplementary Fig. 4. QTRT1/2 complex recognizes and modifies *in-vitro* and native pre-tRNA^{Tyr}.

a SDS PAGE gel showing the purity of the recombinant mTGT. M: molecular weight marker. **b** Time-course data, of [8-^{3}H]-guanine incorporation reactions into m_tRNA^{Tyr}. mQTRT1/2 complex was applied at a concentration of 100 nM and guanine at a concentration of 10 µM with a proportion of 10 % being radioactively labelled. **c** Michaelis-Menten plot derived from data shown in **b**. K_M = 0.23 µM. **d, e, f** [8-^{3}H]-guanine incorporation assay for *in vitro* transcribed tRNA^{Tyr}, pre-tRNA^{Tyr} 1-4 and pre-tRNA^{Tyr} 2-1 respectively. Error bars represent ±SD (n=2, technical replicates, individual datapoints are provided in the source data). **g** Reaction rates and k_{cat} values resulting from the progress curves shown in **d**, **e** and **f**. **h** APB Northern blotting of the samples indicated in Fig. 2b showing the specificity of mQTRT1/2 complex on the indicated pre- and mature tRNA^{Tyr}. Mature tRNA^{Leu} and pre-tRNA^{Leu} 2-1 are used as negative control. A slight unspecific activity compatible with the LC-MS/MS measurements is detectable on pre-tRNA^{Leu} 2-1. The red arrows indicate Q-tRNA shift, the black arrow indicates unmodified pre-tRNA and tRNA. **i** Detection of the covalent intermediates formed between mQTRT1/2 and tRNA^{Tyr}, pre-tRNA^{Tyr} 1-4 or pre-tRNA^{Tyr} 2-1, respectively. The shifted intermediate is detected using Cy5 (tRNA, upper panel) and Coomassie staining (protein, lower panel) and labelled (right).

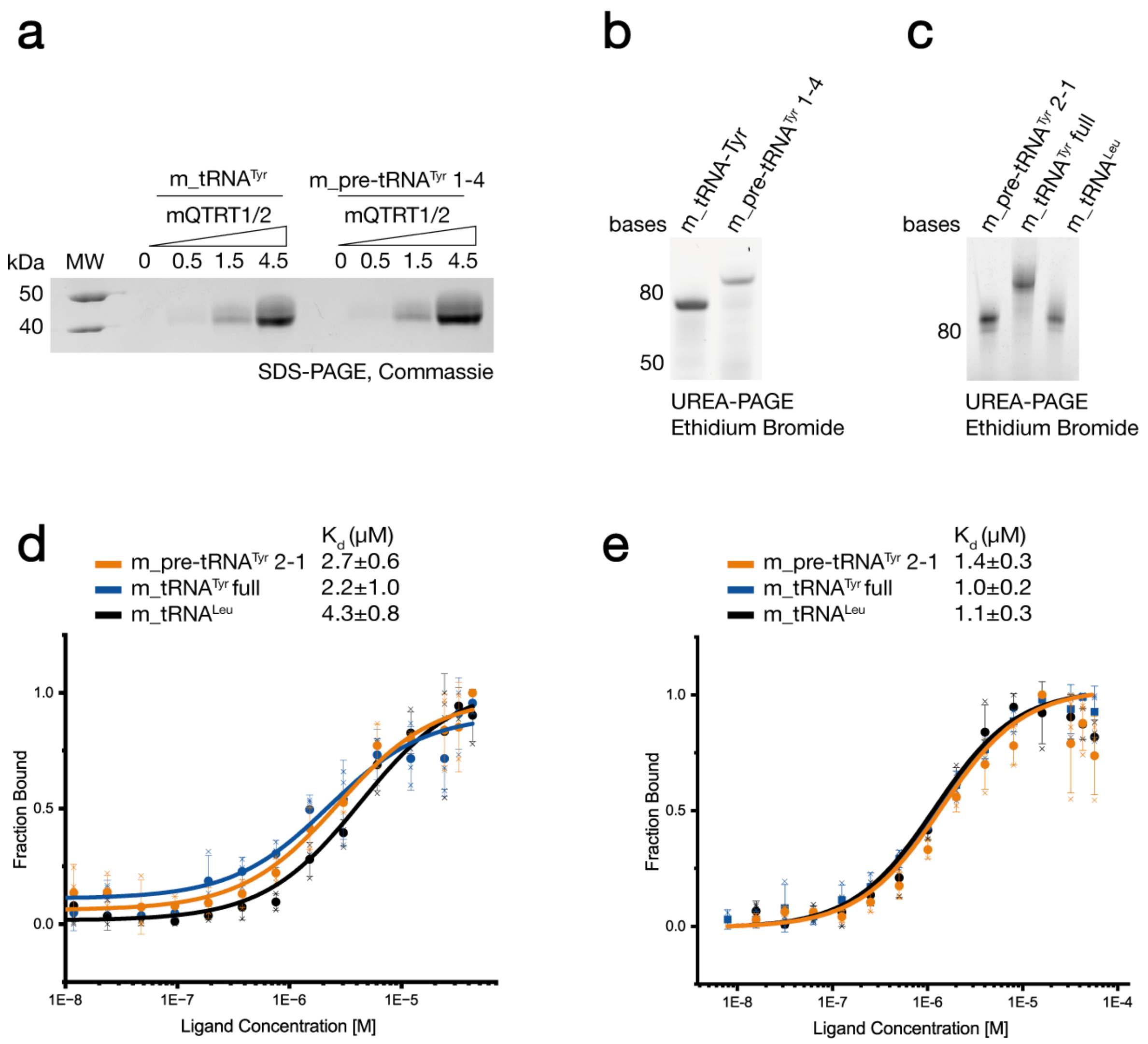

Supplementary Figure 5. Analyses of mQTRT1/2 binding to precursor of tRNA^{Tyr} and tRNA^{Leu}.

a Protein inputs for EMSA analyses of mQTRT1/2 binding to tRNAs in Fig. 3c. Used protein concentrations are shown, whereas tRNA concentrations are kept constant at 200 nM. MW: molecular weight marker. **b** Denaturing UREA-PAGE analysis of tRNAs used in experiment shown in Fig. 3 and Supplementary Fig. 4i. **c** Denaturing UREA-PAGE analysis of tRNAs used in experiment shown in Supplementary Fig. 5d/e. **d** MST analyses of mQTRT1/2 binding to pre-tRNA^{Tyr} 2-1, m_tRNA^{Tyr} full and m_tRNA^{Leu}. Concentrations of mQTRT1/2 and calculated apparent K_d values are given, concentration of tRNA is kept constant at 50 nM. Individual measurements at each concentration are shown with the fitted curve. Error bars represent ±SD (n = 3 biological replicates). **e** MST analyses of mQTRT1/2 binding to pre-tRNA^{Tyr} 2-1, m_tRNA^{Tyr} full and m_tRNA^{Leu}, in the presence of 9dG. Concentrations of mQTRT1/2 and calculated apparent K_d values are given, concentration of tRNA is kept constant at 50 nM. Individual measurements at each concentration are shown with the fitted curve. Error bars represent ±SD (n = 3, biological replicates).

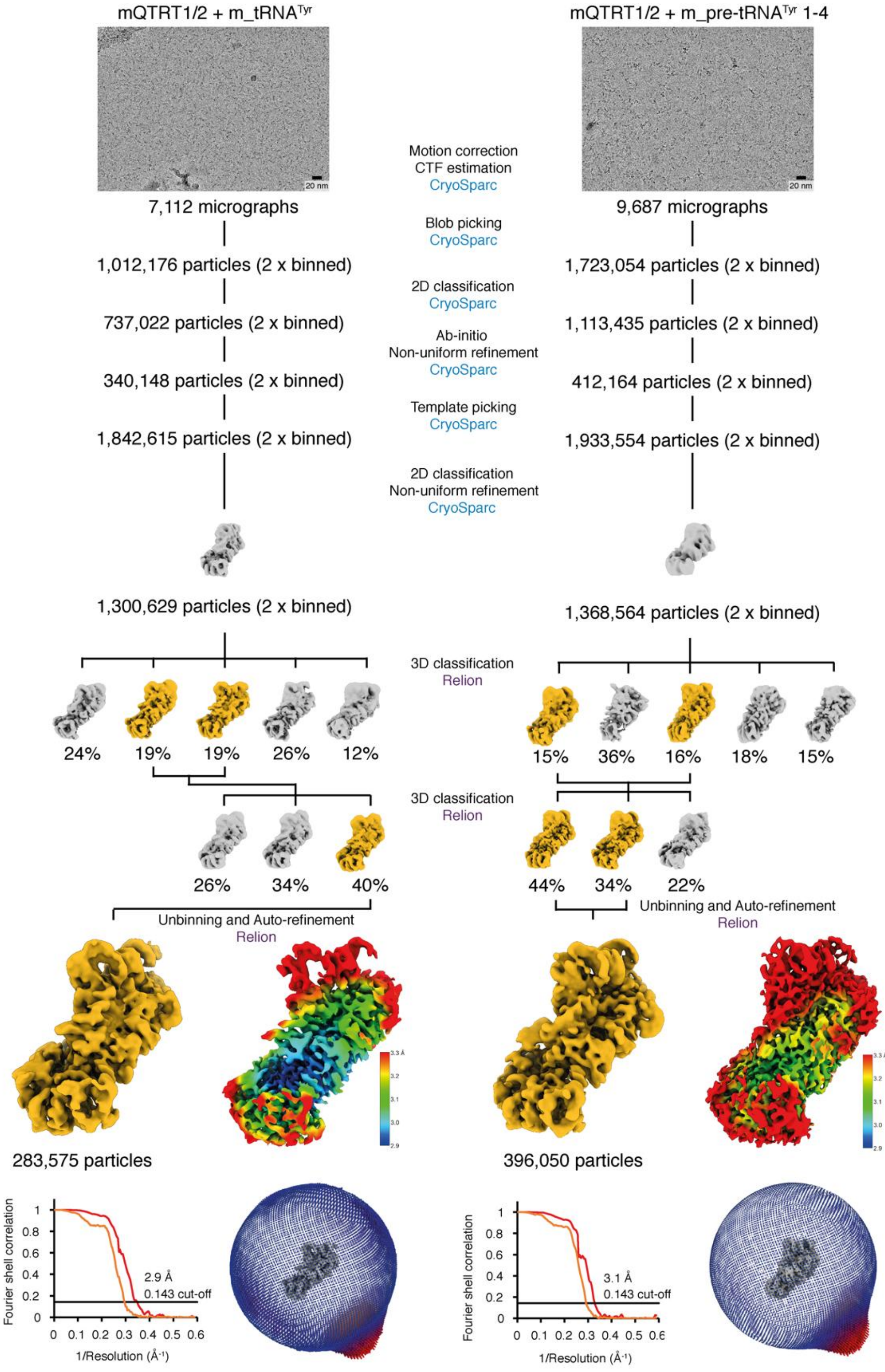

Supplementary Fig. 6. Cryo-EM data processing pipelines.
Rainbow-colored map represents local distribution of resolution. FSC curves: masked (red) and unmasked (orange) are plotted on the bottom. Right panel represents angular views distribution used for final reconstructions for both complexes.

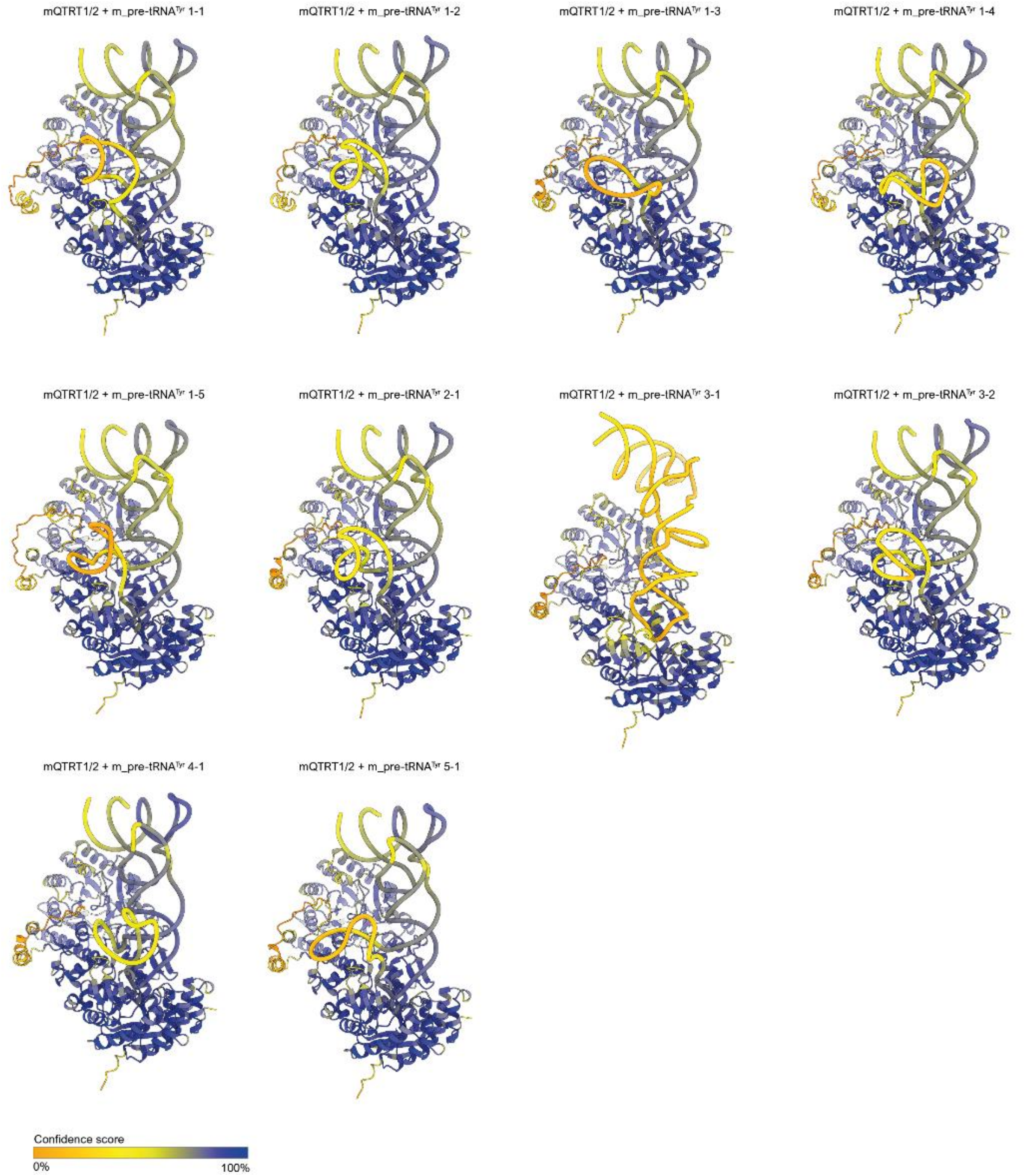

Supplementary Fig. 7. AlphaFold 3 predictions of mQTRT1/2 in complexes with all mouse precursors of tRNA^{Tyr}.

Mouse QTRT1/2 in complexes with: pre-tRNA^{Tyr} 1-1, pre-tRNA^{Tyr} 1-2, pre-tRNA^{Tyr} 1-3, pre-tRNA^{Tyr} 1-4, pre-tRNA^{Tyr} 1-5, pre-tRNA^{Tyr} 2-1, pre-tRNA^{Tyr} 3-1, pre-tRNA^{Tyr} 3-2, pre-tRNA^{Tyr} 4-1, pre-tRNA^{Tyr} 5-1. Models are colored according to their local confidence scores, orange – lowest confidence, blue – highest confidence.

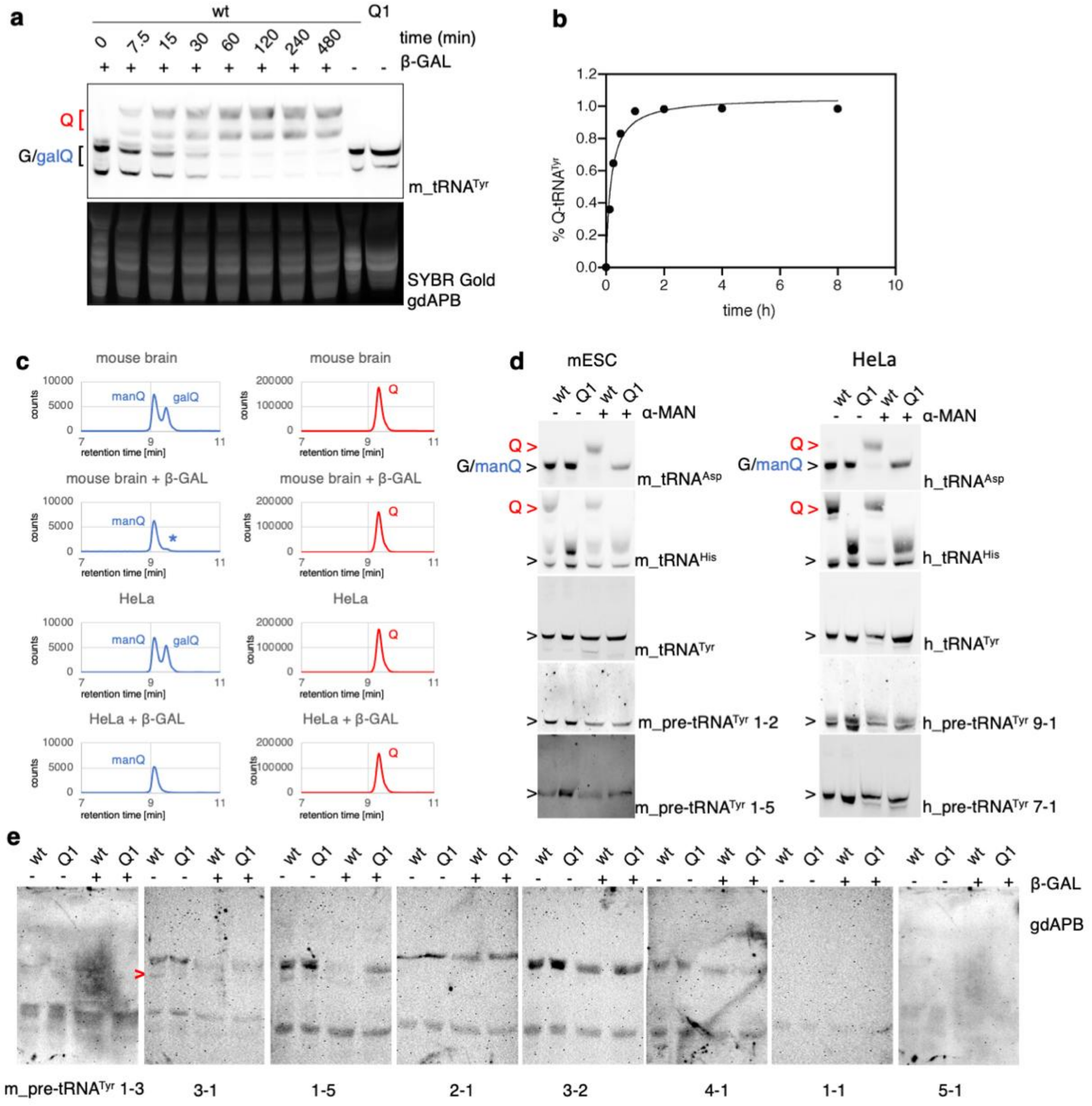

Supplementary Fig. 8. gdAPB Northern blot shows galQ on pre-tRNA^{Tyr}.

a Time course of β1-3,4 Galactosidase (β-GAL) digestion on mouse brain bulk tRNA shows optimal digestion at 1h. Red arrows indicate Q, black arrows indicate unmodified or un-resolved galQ. **b** Quantification in % of the digested galQ revealed by APB Northern blot in **a**. **c** LC-MS/MS is used to detect the depletion of manQ and galQ upon treatment with β-GAL on bulk tRNA isolated from mouse brain and human HeLa cells. Q traces are in red and manQ and galQ in blue. The asterisk indicates an incomplete β-GAL digestion. **d** manQ modification is detected upon digestion with α1-2,3,6 mannosidase (α-MAN) on mature mouse and human RNA^{Asp} on brain tissue (Q1 knockout is shown as negative control) and HeLa cells grown in medium ± q by gdAPB Northern blotting. No manQ can be detected on mature tRNA^{Tyr} and on the shown pre-tRNAs. Red arrows indicate Q, black arrows indicate unmodified or un-resolved man/galQ. **e** gdAPB Northern blotting shows that m_pre-tRNA^{Tyr} 3-1 is also queuosinylated before splicing in mouse brain. The red arrow indicates the shift produced by Q. The analysis completes the data showed in Fig. 4c using probes against all the introns in mouse pre-tRNA^{Tyr}.

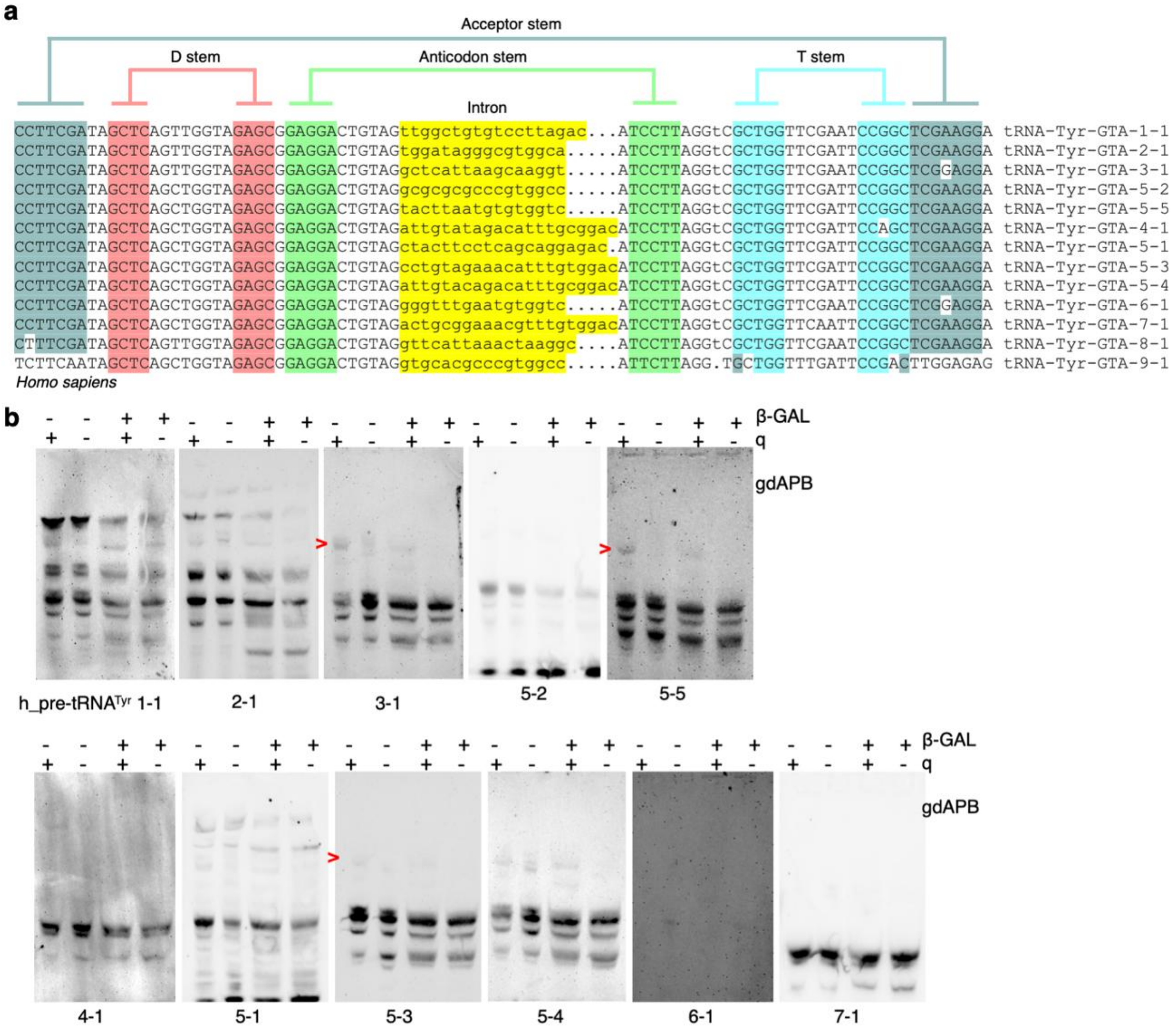

Supplementary Fig. 9. Systematic analysis of the pre-tRNA^{Tyr} modification at position 34 in human.

a Genomic sequence of all the human tRNA^{Tyr} containing an intron according the gtRNA database (https://gtrnadb.ucsc.edu). Specific probes are designed as reverse complementary to the intron sequence in yellow. **b** Q is detected on pre-tRNA^{Tyr} 3-1, 5-5, 5-3 in human HeLa cells grown in medium ± q using gdAPB Northern blotting. The red arrows indicate the shift produced by Q. The analysis completes the data showed in Fig. 5a using probes against all the introns in human pre-tRNA^{Tyr}.

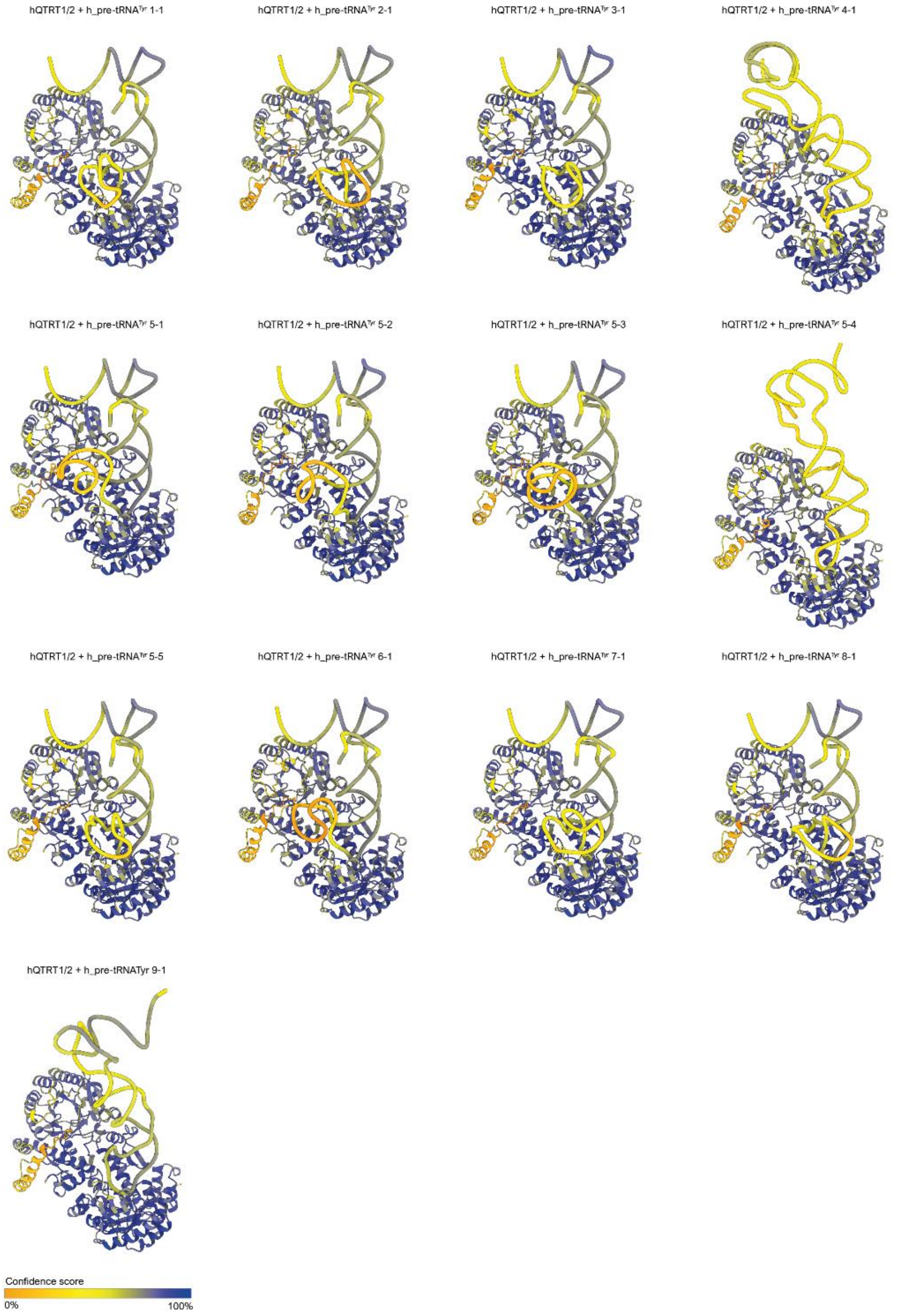

Supplementary Fig. 10. AlphaFold 3 predictions of human QTRT1/2 in complexes with all precursors of tRNA^{Tyr}.

Human QTRT1/2 in complexes with: pre-tRNA^{Tyr} 1-1, pre-tRNA^{Tyr} 2-1, pre-tRNA^{Tyr} 3-1, pre-tRNA^{Tyr} 4-1, pre-tRNA^{Tyr} 5-1, pre-tRNA^{Tyr} 5-2, pre-tRNA^{Tyr} 5-3, pre-tRNA^{Tyr} 5-4, pre-tRNA^{Tyr} 5-5, pre-tRNA^{Tyr} 6-1, pre-tRNA^{Tyr} 7-1, pre-tRNA^{Tyr} 8-1, pre-tRNA^{Tyr} 9-1. Models are coloured according to their local confidence scores, orange – lowest confidence, blue – highest confidence.

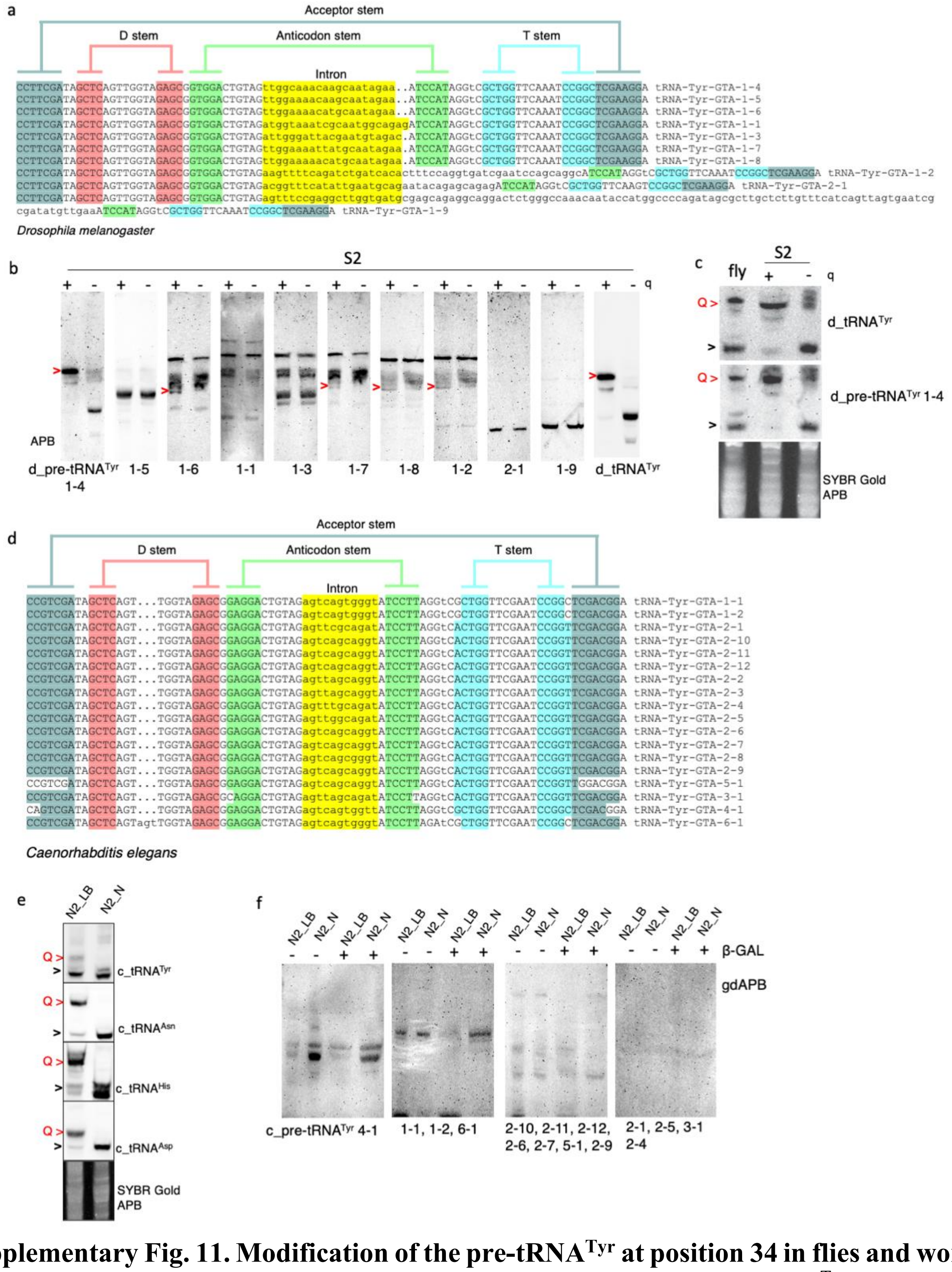

Supplementary Fig. 11. Modification of the pre-tRNA^{Tyr} at position 34 in flies and worms. **a** and **d** Genomic sequences of all the *D. melanogaster* and *C. elegans* tRNA^{Tyr} containing an intron according the gtRNA database (https://gtrnadb.ucsc.edu). Specific probes are designed reverse complementary to the intron sequence in yellow. **b** Q is detected on pre-tRNA^{Tyr} 1-6, 1-7, 1-8 and 1-2 in *D. melanogaster* S2 cells grown in medium ± q (queuine) using gdAPB Northern blot. The analysis completes the data showed in Fig. 5b using probes against all the introns in *D. melanogaster* pre-tRNA^{Tyr}. **c** Q modification is detected on d_pre-tRNA^{Tyr} 1-4 in *D. melanogaster* adult flies. **e** Queuosinylation of mature c_ tRNA^{Tyr}, ^{Asn}, ^{His}, ^{Asp} is present in *C. elegans* grown in peptone rich medium (LB) and is not detected in standard NGM plates (N). **f** The indicated probes for pre-tRNA^{Tyr} in *C. elegans* did not detect modification.

The red arrows indicate the shift produced by Q, the black arrow indicates unmodified pre-tRNA and tRNA. LB: peptone rich medium, N: NGM plates.

Supplementary tables

Supplementary Table 1. Cryo-EM data collection, refinement and validation statistics.

	mQTRT1/2 + m_tRNA^{Tyr} PDB: 9HN7; EMD-52308; EMPIAR-XXX	mQTRT1/2 + m_pre-tRNA^{Tyr}1-4 PDB:9HN9; EMD-52309; EMPIAR-XXX
**Data collection and processing**		
Magnification	105,000x	105,000x
Voltage (keV)	300	300
Electron exposure (e-/ Å^2)	40	40
Defocus range (µM)	-0.6 to -1.5	-0.6 to -1.5
Pixel size (Å)	0.84	0.84
Symmetry imposed	C1	C1
Initial particle images (no.)	1,842,615	1,933,554
Final particle images (no.)	283,575	396,050
Map resolution (Å)	2.9	3.1
FSC threshold	0.143	0.143
Map resolution range (Å)	2.87 - 4.83	3.00 - 4.85
**Refinement**		
Initial model used	7OV9 (QTRT1/2) and Alphafold3 (tRNA)	7OV9 (QTRT1/2) and Alphafold3 (tRNA)
FSC threshold	0.143	0.143
B-factors (min/max/mean)		
Protein	84.91/179.10/123.46	78.78/257.33/145.05
tRNA	114.98/240.64/168.32	93.93/575.09/236.54
9dG	138.75/138.75/138.75	157.36/162.80/159.71
**Model composition**		
Non-hydrogen atoms	6962	6897
Protein residues	705	705
Nucleotide residues	76	73
Ligands	9dG: 1	9dG: 1
**Root-mean-sqare-deviations (RMSDs)**		
Bond lengths (Å)	0.002	0.005
Bond angles (°)	0.516	1.118
**Validation**		
MolProbity score	1.74	1.97
Clashscore	13.66	10.66
Poor rotamers (%)	0.00	0.00
**Ramachandran**		
Favored (%)	97.54	93.63
Allowed (%)	2.46	6.37
Disallowed (%)	0.00	0.00
CC volume (%)	0.47	0.60

Supplementary Table 2. Q status for pre-tRNA across species.

pre-tRNA^{Tyr} detected to be queuosinylated and galactosylated in the indicated species and conditions. N/A not tested, pre-tRNA not expressed are represented with gray cells.

M. musculus **mESCs**	Q	galQ	locus	expression % of pre-tRNATyr
tRNA-Tyr-GTA-1-2		√	chr13:23426505-23426592 (+)	26
tRNA-Tyr-GTA-1-3		N/A	chr13:23427095-23427180 (-)	4
tRNA-Tyr-GTA-1-4	√	√	chr13:23432990-23433077 (-)	12
tRNA-Tyr-GTA-3-1		N/A	chr3:19628782-19628870 (+)	26
tRNA-Tyr-GTA-1-5		N/A	chr13:23467076-23467165 (+)	13
tRNA-Tyr-GTA-2-1	√	N/A	chr5:30888083-30888171 (+)	1
tRNA-Tyr-GTA-3-2		N/A	chr14:51089489-51089578 (+)	1
tRNA-Tyr-GTA-4-1		N/A	chr3:19628355-19628447 (+)	9
tRNA-Tyr-GTA-1-1		N/A	chr13:23425646-23425734 (-)	5
tRNA-Tyr-GTA-5-1	√	N/A	chr13:23428738-23428826 (-)	2

M. musculus **mouse brain**	Q	galQ	expression %of pre-tRNATyr
tRNA-Tyr-GTA-1-2		√	22
tRNA-Tyr-GTA-1-3			7
tRNA-Tyr-GTA-1-4	√	√	10
tRNA-Tyr-GTA-3-1	√		28
tRNA-Tyr-GTA-1-5			9
tRNA-Tyr-GTA-2-1			2
tRNA-Tyr-GTA-3-2			2
tRNA-Tyr-GTA-4-1			11
tRNA-Tyr-GTA-1-1			4
tRNA-Tyr-GTA-5-1			3

H. sapiens **HeLa**	Q	galQ	locus
tRNA-Tyr-GTA-1-1			chr6:26568858-26568948 (+)
tRNA-Tyr-GTA-2-1			chr2:27050782-27050870 (+)
tRNA-Tyr-GTA-3-1	√		chr6:26577104-26577192 (+)
tRNA-Tyr-GTA-5-2			chr8:66113988-66114076 (+)
tRNA-Tyr-GTA-5-5	√		chr14:20683273-20683361 (+)
tRNA-Tyr-GTA-4-1			chr14:20657464-20657557 (-)
tRNA-Tyr-GTA-5-1			chr8:66113367-66113459 (+)
tRNA-Tyr-GTA-5-3	√		chr14:20653099-20653192 (-)
tRNA-Tyr-GTA-5-4			chr14:20663192-20663285 (-)
tRNA-Tyr-GTA-6-1			chr6:26594874-26594962 (+)
tRNA-Tyr-GTA-7-1			chr14:20659958-20660051 (-)
tRNA-Tyr-GTA-8-1		√	chr6:26575570-26575659 (+)
tRNA-Tyr-GTA-9-1		√	chr8:65697297-65697384 (-)

C. elegans	Q	galQ	locus
tRNA-Tyr-GTA-2-10			chrX:16208825-16208908 (-)
tRNA-Tyr-GTA-2-11			chrX:16209228-16209311 (-)
tRNA-Tyr-GTA-2-12			chrX:16209769-16209852 (-)
tRNA-Tyr-GTA-2-6			chrX:865389-865472 (-)
tRNA-Tyr-GTA-2-7			chrX:12665228-12665311 (+)
tRNA-Tyr-GTA-5-1			chrX:13284766-13284849 (-)
tRNA-Tyr-GTA-2-9			chrX:16208263-16208346 (-)
tRNA-Tyr-GTA-2-2			chrIII:535382-535465 (+)
tRNA-Tyr-GTA-2-3			chrIII:13224566-13224649 (+)
tRNA-Tyr-GTA-1-1			chrV:8247481-8247564 (+)
tRNA-Tyr-GTA-1-2			chrV:15480694-15480777 (+)
tRNA-Tyr-GTA-6-1			chrV:9412205-9412291 (-)
tRNA-Tyr-GTA-4-1			chrII:9211804-9211887 (-)
tRNA-Tyr-GTA-2-8	√		chrX:13264683-13264766 (-)
tRNA-Tyr-GTA-2-1			chrI:6135830-6135913 (+)
tRNA-Tyr-GTA-2-5			chrV:17542446-17542529 (-)
tRNA-Tyr-GTA-3-1			chrV:17317879-17317962 (-)
tRNA-Tyr-GTA-2-4			chrV:14689532-14689615 (+)

D. melanogaster **S2**	Q	galQ	locus
tRNA-Tyr-GTA-1-4	√		chr3R:8642333-8642425 (-)
tRNA-Tyr-GTA-1-5			chr3R:8642701-8642793 (-)
tRNA-Tyr-GTA-1-6	√		chr3R:8668116-8668208 (-)
tRNA-Tyr-GTA-1-1			chrX:21304441-21304535 (-)
tRNA-Tyr-GTA-1-3			chr2L:2462593-2462686 (+)
tRNA-Tyr-GTA-1-7	√		chr3R:8668612-8668705 (-)
tRNA-Tyr-GTA-1-8	√		chr3R:8669081-8669174 (-)
tRNA-Tyr-GTA-1-2	√		chr2L:2459924-2460044 (-)
tRNA-Tyr-GTA-2-1			chrX:21304713-21304820 (+)
tRNA-Tyr-GTA-1-9			chr2L:7711476-7711661 (+)

D. melanogaster **fly**	Q	galQ
tRNA-Tyr-GTA-1-4	√	
tRNA-Tyr-GTA-1-5	N/A	
tRNA-Tyr-GTA-1-6	N/A	
tRNA-Tyr-GTA-1-1	N/A	
tRNA-Tyr-GTA-1-3	N/A	
tRNA-Tyr-GTA-1-7	N/A	
tRNA-Tyr-GTA-1-8	N/A	
tRNA-Tyr-GTA-1-2	N/A	
tRNA-Tyr-GTA-2-1	N/A	
tRNA-Tyr-GTA-1-9	N/A	

Supplementary Methods

mESCs differentiantion

Differentiation was performed using the hanging droplet method[1]. As described, mESC medium without LIF and with 250 nM retinoic acid was used for the neuronal differentiation. RT-qPCR was used to validate stemness and neuronal differentiation. Briefly: 1 μg total RNA was used for reverse transcription using 100 U M-MLV Reverse Transcriptase (Promega), 4 μM Random Hexamer Primers (Thermo Fisher Scientific), 20 U RNasin Ribonuclease Inhibitor (Promega) and dNTPs (1 mM each) for 1 h at 37 °C. PCR reactions were performed using the Powerup SYBR Green Master Mix (Thermo Fisher Scientific) with the following primers:ActBF: AGGTGTGATGGTGGAATGG ActBR: GGTTGGCCTTAGGGTTCAGG

Oct4F: TGCGAGGGATGGCATACTG Oct4R: GCACAGGGCTCAGAGGAGG

NestinF: AGAGTCGCTTAGAGGTGCAG NestinR: AGCCACTTCCAGACTAAGGG

Cytosolic and nuclear fractionation

10^5 -10^7 cells were lysed using in 20 mM Tris-HCl, pH 7.5, 5 mM $MgCl_2$, 150 mM NaCl, 1% Triton X-100, 1 mM DTT and complete protease inhibitor (Roche) by gently tumbling at 4° C for 10 minutes. The samples were then centrifuged at 4° C for 10 minutes. The pellets were resuspended in 0.1% SDS in 10 mM Tris-HCl, pH 8.5 and sonicated to solubilize protein and RNA. The pellets were used to obtain nuclear fractions, while the supernatants were collected for cytosolic fractions. Proportional nuclear and cytosolic fractions were loaded into a 10% SDS-PAGE gel for conventional Western blotting analysis (antibodies: eIF3A 1:1000 Cell Signaling #3411, Lamin A/C 1:1000 Cell Signaling #4777S). In parallel, nuclear and cytosolic fractions were mixed with 10 mM Tris-HCl, pH 7.5; 350 mM NaCl; 10 mM EDTA; 1% SDS; 42% urea, heated at 65°C for 10 minutes, and RNA was extracted using Phenol:Chloroform:Isoamyl Alcohol, followed by an additional purification with TRIzol

(Invitrogen) according the manufacturer's instructions for a subsequent Northern blotting analysis.

Cloning, expression and purification of human TSEN complex

The TSEN subunits TSEN15 (UniProtKB Q8WW01), TSEN34 (UniProtKB Q9BSV6), TSEN54 (UniProtKB Q7Z6J9) and TSEN2 (UniProtKB Q8NCE0) were cloned into MultiBac or pFastBac vectors. The resulting constructs were pIDK-TSEN34 (donor), pIDK-6xHis-TEV-TSEN15 (donor), pAceBac-TSEN54 (acceptor) and pFastBac-6xHis-TEV-TSEN2. Acceptor and donor vectors were merged by Cre-mediated recombination. Recombinant baculoviral BACs for human TSEN subunits were generated via Tn7 transposition in *Escherichia coli* DH10EMBacY cells (Geneva Biotech).

Production of baculoviruses and expression of human TSEN complex was performed in *Spodoptera frugiperda* (Sf) 21 cells. Sf21 cells (500 mL suspension) were infected with 1% (v/v) V1 baculovirus supernatant at a density of 0.9−1.1×10 cells/mL for 72 hours after proliferation arrest. Protein expression was assessed by SDS-PAGE using Coomassie staining. Cell pellets were collected by centrifugation (500 g, 10 min), flash-frozen in liquid nitrogen, and stored at -80°C. Proteins were extracted using a 110L Microfluidizer (Microfluidics, USA) in lysis buffer (50 mM Tris pH 8.0, 300 mM NaCl, 20 mM imidazole, 10% glycerol, 1 mM TCEP, and 500 µL EDTA-free protease inhibitor per 100 mL expression volume). Cleared lysates were loaded with a Superloop 150 (Cytiva) onto a 5 mL Ni-NTA column (Cytiva) and protein elution was performed using elution buffer (50 mM Tris pH 8.0, 300 mM NaCl, 500 mM imidazole, 10% glycerol, 1 mM TCEP). TSEN fractions were pooled and incubated with Tobacco Etch Virus (TEV) protease (1:100 w/w) at 4°C overnight and then applied back onto a 5ml Ni-NTA column (Cytiva) for the removal of the cleaved 6xHis tag peptide. Protein was concentrated and separated according to size by gelfiltration using a HiLoad 16/600 Superdex 200 pg column (Cytiva) with a buffer consisting of 25 mM Tris pH 7.5, 100 mM NaCl, 10%

glycerol 1 mM TCEP. TSEN complex-containing fractions were pooled, concentrated using an Amicon ultrafiltration device (30 kDa MWCO, Sigma) and flash-frozen in liquid nitrogen for storage at -80°C.

TSEN Activity Assay

In vitro transcribed tRNA (HiScribe® T7 High Yield RNA Synthesis Kit, NEB) was purified from urea-PAGE gel. The tRNA was unfolded at 90°C for 1 min, then refolded by cooling to ~40°C. TSEN activity assays were performed by incubating 1 µM TSEN complex with 1 µM *in vitro* transcribed tRNA or 150 µM bulk tRNA (wildtype/Q1 mESC) in 50 mM HEPES-NaOH, pH 7.4; 100 mM NaCl; 4 mM $MgCl_2$; 0.5 mM TCEP (20 µL reaction) at 37°C. At indicated time points, 3 µL aliquots were quenched in 30 µL stop solution (10 M urea, 0.1% SDS, 1 mM EDTA, 0.05% xylene cyanol, 0.05% bromophenol blue). Reaction products were analyzed via 15% urea-PAGE using SYBR™ Gold staining (Invitrogen, Thermo Fisher) or Northern blotting.

[8-^{3}H]-guanine incorporation assay

The [8-^{3}H]-guanine incorporation assay for TGT was performed similar as previously described [2]. 38 µL of a TGT-containing solution were added to 38 µL of a solution containing the specific tRNAs and 10% - [8-^{3}H]-guanine (American Radiolabelled Chemicals). Before combining the solutions, both were preheated at 37 °C and held at that temperature during the reaction time. A 15 µL aliquot is pipetted onto a glass fiber filter (Sartorius) after 4, 8, 12, and 21 minutes and subsequently washed for 10 min in 10% TCA, twice in 5% TCA and for 20 min in 96% ethanol. After drying for 45 min at 60°C and adding a scintillation cocktail (ROTISZINT® Filter, Carl Roth), the filters were measured in a scintillation counter (Hidex 300 SL). To convert the measured cpm values into a concentration, a calibration line with different concentrations of radiolabelled guanine was created. The final concentrations of the components were 10 µM 10% - [8-^{3}H]-guanine and 100 nM TGT. For determining Michaelis-

Menten kinetics, the tRNA concentrations were varied from 0.26 to 15 µM. In cases where no Michaelis-Menten kinetics could be determined, a tRNA concentration of 15 µM was used to determine an approximate k_{cat} and aliquots were taken over the course of an hour.

Covalent intermediate test

Purified murine QTRT1/2 complex was mixed with the respective *in vitro* transcribed tRNA (labelled with Cy5-cytosine on the stage of *in vitro* transcription) at a molar ratio of 1:2, in buffer containing 20 mM HEPES pH 7.5, 100 mM NaCl, 1 mM $MgCl_2$, 2 mM DTT and 9dG in the final concentration of 100 µM. Samples were incubated at 25 °C for 60 min, then denatured by incubation at 95 °C for 5 min. Samples were resolved using SDS-Page or Bis-Tris 4-12% gradient gels (Thermo Fisher Scientific). Gels were scanned using a ChemiDoc MP Imaging System (BioRad), equipped with Cy5 filter and analysed in Image Lab Software (BioRad). After detection of fluorescence signals, gels were stained with Coomassie brilliant blue and visualised using ChemiDoc MP Imaging System (BioRad).

Supplementary References

1. Witteveldt J, Macias S. Differentiation of Mouse Embryonic Stem Cells to Neuronal Cells Using Hanging Droplets and Retinoic Acid. *Bio Protoc* **9**, e3417 (2019).

2. Sebastiani M*, et al.* Structural and Biochemical Investigation of the Heterodimeric Murine tRNA-Guanine Transglycosylase. *ACS Chem Biol* **17**, 2229-2247 (2022).
