# Supplementary material for: Queuosine is incorporated into precursor tRNA before splicing
Source: Nat Commun. 2025 Jul 31;16:7044. doi: 10.1038/s41467-025-62220-z (PMC12313893; doi:10.1038/s41467-025-62220-z)
Supplement: Supplementary file 2 — Description of Additional Supplementary Files [file 41467_2025_62220_MOESM2_ESM.pdf]

Description of Additional Supplementary Files

Supplementary Data 1: *In vitro* transcribed tRNA sequences and probes used in Northern blots
