# Supplementary material for: Queuosine is incorporated into precursor tRNA before splicing
Source: Nat Commun. 2025 Jul 31;16:7044. doi: 10.1038/s41467-025-62220-z (PMC12313893; doi:10.1038/s41467-025-62220-z)
Supplement: Supplementary file 4 — Reporting Summary [file 41467_2025_62220_MOESM4_ESM.pdf]

nature portfolio

Corresponding author(s): Francesca Tuorto

Last updated by author(s): Jul 4, 2025

Reporting Summary

Nature Portfolio wishes to improve the reproducibility of the work that we publish. This form provides structure for consistency and transparency in reporting. For further information on Nature Portfolio policies, see our Editorial Policies and the Editorial Policy Checklist.

Statistics

For all statistical analyses, confirm that the following items are present in the figure legend, table legend, main text, or Methods section.

n/a	Confirmed	
☐	☒	The exact sample size (*n*) for each experimental group/condition, given as a discrete number and unit of measurement
☐	☒	A statement on whether measurements were taken from distinct samples or whether the same sample was measured repeatedly
☐	☒	The statistical test(s) used AND whether they are one- or two-sided<br>*Only common tests should be described solely by name; describe more complex techniques in the Methods section.*
☐	☒	A description of all covariates tested
☐	☒	A description of any assumptions or corrections, such as tests of normality and adjustment for multiple comparisons
☐	☒	A full description of the statistical parameters including central tendency (e.g. means) or other basic estimates (e.g. regression coefficient) AND variation (e.g. standard deviation) or associated estimates of uncertainty (e.g. confidence intervals)
☒	☐	For null hypothesis testing, the test statistic (e.g. *F*, *t*, *r*) with confidence intervals, effect sizes, degrees of freedom and *P* value noted<br>*Give P values as exact values whenever suitable.*
☒	☐	For Bayesian analysis, information on the choice of priors and Markov chain Monte Carlo settings
☒	☐	For hierarchical and complex designs, identification of the appropriate level for tests and full reporting of outcomes
☒	☐	Estimates of effect sizes (e.g. Cohen's *d*, Pearson's *r*), indicating how they were calculated

Our web collection on statistics for biologists contains articles on many of the points above.

Software and code

Policy information about availability of computer code

Data collection	For Cryo-EM structures, we used EPU v. 2.10.0.1941REL (Thermo Fisher).
Data analysis	For Cryo-EM structure analysis, we used ChimeraX 1.2.5, CryoSPARC 3.3.0, DeepEMhancer 0.14, Durchlichtelektronenmikroskopiebilddatenentzerrungswerkzeug 1.0.9, Excel 365, ISOLDE 1.4, Namdinator 2.12, Phenix 1.19.2-4158, PyMOL 1.7, Relion 3.1, WinCOOT 0.9.7 EL.<br>For data analysis, we used E-CRISP (http://www.e-crisp.org/), Fiji 2.15.0, GraphPad Prism 8.4.3, OriginPro 2018.<br>For Microscale thermophoresis data analysis was performed using MO. Control software (NanoTemper Technologies) and MO. AffinityAnakysis (Nano Temper Technologies).<br>Northern blot membranes and gels were scanned using ChemiDoc MP Imaging System (BioRad) and analyzed in Image Lab Software (BioRad) or Adobe Photoshop 22.2.0.<br>QTRT1/Qtrt2-tRNA models predicted with AlphaFold 3- ChimeraX (rigid-body fit), then flexibly fitted using ISOLDE (maintaining all secondary structures) and manually curated using WinCoot. Models were further refined and validated in Phenix.<br>Diagrams and statistics were performed using GraphPad Prism 8.4.3.<br>Chemical structure were designed using Chem Draw 20.0.

For manuscripts utilizing custom algorithms or software that are central to the research but not yet described in published literature, software must be made available to editors and reviewers. We strongly encourage code deposition in a community repository (e.g. GitHub). See the Nature Portfolio guidelines for submitting code & software for further information.

Data

Policy information about availability of data

All manuscripts must include a data availability statement. This statement should provide the following information, where applicable:

- Accession codes, unique identifiers, or web links for publicly available datasets
- A description of any restrictions on data availability
- For clinical datasets or third party data, please ensure that the statement adheres to our policy

The atomic coordinates and cryo-EM maps have been deposited and validated in the EMData Bank (EMDB) and the Protein Data Bank (PDB) under the following accession codes – mouse QTRT1/2 with mature tRNATyr (PDB ID 9HN7; EMD-52308) and mouse QTRT1/2 with precursor tRNATyr 1-4 (PDB ID 9HN9; EMD-52309). All entries will be publicly released upon publication.
Uncropped and unprocessed scans of the gels and Northern blots are provided in the Source Data file.

Research involving human participants, their data, or biological material

Policy information about studies with human participants or human data. See also policy information about sex, gender (identity/presentation), and sexual orientation and race, ethnicity and racism.

Reporting on sex and gender	*Use the terms sex (biological attribute) and gender (shaped by social and cultural circumstances) carefully in order to avoid confusing both terms. Indicate if findings apply to only one sex or gender; describe whether sex and gender were considered in study design; whether sex and/or gender was determined based on self-reporting or assigned and methods used.*<br>*Provide in the source data disaggregated sex and gender data, where this information has been collected, and if consent has been obtained for sharing of individual-level data; provide overall numbers in this Reporting Summary. Please state if this information has not been collected.*<br>*Report sex- and gender-based analyses where performed, justify reasons for lack of sex- and gender-based analysis.*
Reporting on race, ethnicity, or other socially relevant groupings	*Please specify the socially constructed or socially relevant categorization variable(s) used in your manuscript and explain why they were used. Please note that such variables should not be used as proxies for other socially constructed/relevant variables (for example, race or ethnicity should not be used as a proxy for socioeconomic status).*<br>*Provide clear definitions of the relevant terms used, how they were provided (by the participants/respondents, the researchers, or third parties), and the method(s) used to classify people into the different categories (e.g. self-report, census or administrative data, social media data, etc.)*<br>*Please provide details about how you controlled for confounding variables in your analyses.*
Population characteristics	*Describe the covariate-relevant population characteristics of the human research participants (e.g. age, genotypic information, past and current diagnosis and treatment categories). If you filled out the behavioural & social sciences study design questions and have nothing to add here, write "See above."*
Recruitment	*Describe how participants were recruited. Outline any potential self-selection bias or other biases that may be present and how these are likely to impact results.*
Ethics oversight	*Identify the organization(s) that approved the study protocol.*

Note that full information on the approval of the study protocol must also be provided in the manuscript.

Field-specific reporting

Please select the one below that is the best fit for your research. If you are not sure, read the appropriate sections before making your selection.

- [x] Life sciences
- [ ] Behavioural & social sciences
- [ ] Ecological, evolutionary & environmental sciences

For a reference copy of the document with all sections, see nature.com/documents/nr-reporting-summary-flat.pdf

Life sciences study design

All studies must disclose on these points even when the disclosure is negative.

Sample size	Sample size for each experiment is indicated in the figure legend. Mouse tissue Northern blots were performed comparing 3 biological replicates per genotype, 2 female and 1 male mice, with matched sex and age. No statistical sample size calculation was performed to predetermine sample sizes. Sample sizes were based on previous experience with the experiments performed. All available mice were used to provide sufficient statistical power in the mouse experiments.<br>For cryo-EM analyses, sample size for each experiment is indicated in the figure legend and was chosen so that high resolution structures are constructed.
Data exclusions	For cryo-EM analyses, several iterations of reference-free 2D class averaging and unsupervised 3D classification were used to remove suboptimal particles. For the other experiments no data were excluded.
Replication	All experimental findings were successfully reproduced in multiple independent experiments and orthogonal approaches, which are indicated

in the results, in figure legends and In The Statistic and Reproducibility paragraph of the Methods section.

Randomization	Mice were chosen randomly among litter-mates and were allocated to their respective groups based on their genotype and sex (control & Q1, female & male). C. elegans and D. melanogaster were collected in stock groups with randomized individuals.
Blinding	No specific blinding was used for the experiments presented in the study. Blinding is not relevant to Northern blotting, LC/MS-MS, cryo-EM and biophysical analyses in this study.

Reporting for specific materials, systems and methods

We require information from authors about some types of materials, experimental systems and methods used in many studies. Here, indicate whether each material, system or method listed is relevant to your study. If you are not sure if a list item applies to your research, read the appropriate section before selecting a response.

Materials & experimental systems

n/a	Involved in the study	
☐	☒	Antibodies
☐	☒	Eukaryotic cell lines
☒	☐	Palaeontology and archaeology
☐	☒	Animals and other organisms
☒	☐	Clinical data
☒	☐	Dual use research of concern
☒	☐	Plants

Methods

n/a	Involved in the study	
☒	☐	ChIP-seq
☒	☐	Flow cytometry
☒	☐	MRI-based neuroimaging

Antibodies

Antibodies used	eIF3A 1:1000 Cell Signalling #3411; Lamin AC 1:1000 Cell Signalling #4777S
Validation	eIF3A Cell Signalling #3411: applications: Western Blotting, reactivity:Mouse. This antibody has been validated in multiple studies including PMID: 38514606.<br>Lamin AC Cell Signalling #4777S applications: Western Blotting, reactivity:Mouse. This antibody has been validated in multiple studies including PMID: 40269168.

Eukaryotic cell lines

Policy information about cell lines and Sex and Gender in Research

Cell line source(s)	Male E14 primary mouse embryonic stem cell line (mESC) (background 129/Ola) from Transgenic Mouse Facility of DKFZ.. Q1 and Q2 clones were derived from the wild type cell lines.<br>HeLa cell line from ATCC.<br>Drosophila melanogaster Schneider's 2 cell line from Aurelio Teleman Laboratory (DKFZ).
Authentication	For Human HeLa cell line, the Human Cell Line Authentication Test was performed. For Drosophila S2 cell line, the Multiplex Cell Contamination Test was performed.<br>The mESC were successfully used to produce chimeras and germ line transmission.
Mycoplasma contamination	All cell lines are tested negative for mycoplasma contamination.
Commonly misidentified lines (See ICLAC register)	No commonly misidentified cell line was used in this study.

Animals and other research organisms

Policy information about studies involving animals; ARRIVE guidelines recommended for reporting animal research, and Sex and Gender in Research

Laboratory animals	Species: Mus musculus; strains: C57Bl/6J, C57BL/6J-Qtrt1em1Tuo; adult female and male at an age of 2 months.The husbandry of mice was performed at Mannheim Faculty of Medicine, University of Heidelberg with 12:12 light:dark cycles at standard housing temperatures of 18–23°C. The animal tissue dissection was carried out in strict compliance with national and international guidelines for the Care and Use of Laboratory Animals (Regierungspräsidium Karlsruhe, Germany).<br>Species: Caenorhabditis elegans; strain: N2.<br>Species: Drosophila melanogaster; strain: w1118; sex is mixed , males and females.
Wild animals	The study did not involve wild animals.

Reporting on sex	*Indicate if findings apply to only one sex; describe whether sex was considered in study design, methods used for assigning sex. Provide data disaggregated for sex where this information has been collected in the source data as appropriate; provide overall numbers in this Reporting Summary. Please state if this information has not been collected. Report sex-based analyses where performed, justify reasons for lack of sex-based analysis.*
Field-collected samples	*For laboratory work with field-collected samples, describe all relevant parameters such as housing, maintenance, temperature, photoperiod and end-of-experiment protocol OR state that the study did not involve samples collected from the field.*
Ethics oversight	Animal experiments were conducted in strict compliance with national and international guidelines for the Care and Use of Laboratory Animals and was approved by the local government.

Note that full information on the approval of the study protocol must also be provided in the manuscript.

Plants

Seed stocks	*Report on the source of all seed stocks or other plant material used. If applicable, state the seed stock centre and catalogue number. If plant specimens were collected from the field, describe the collection location, date and sampling procedures.*
Novel plant genotypes	*Describe the methods by which all novel plant genotypes were produced. This includes those generated by transgenic approaches, gene editing, chemical/radiation-based mutagenesis and hybridization. For transgenic lines, describe the transformation method, the number of independent lines analyzed and the generation upon which experiments were performed. For gene-edited lines, describe the editor used, the endogenous sequence targeted for editing, the targeting guide RNA sequence (if applicable) and how the editor was applied.*
Authentication	*Describe any authentication procedures for each seed stock used or novel genotype generated. Describe any experiments used to assess the effect of a mutation and, where applicable, how potential secondary effects (e.g. second site T-DNA insertions, mosiacism, off-target gene editing) were examined.*
